# The Signature of Southern Hemisphere Atmospheric Circulation Patterns in Antarctic Precipitation

**DOI:** 10.1002/2017GL075998

**Published:** 2017-11-18

**Authors:** Gareth J. Marshall, David W. J. Thompson, Michiel R. van den Broeke

**Affiliations:** ^1^ British Antarctic Survey Natural Environment Research Council Cambridge UK; ^2^ Department of Atmospheric Science Colorado State University Fort Collins CO USA; ^3^ Institute for Marine and Atmospheric Research Utrecht University Utrecht Netherlands

**Keywords:** Antarctica, precipitation, circulation, climate, mass balance

## Abstract

We provide the first comprehensive analysis of the relationships between large‐scale patterns of Southern Hemisphere climate variability and the detailed structure of Antarctic precipitation. We examine linkages between the high spatial resolution precipitation from a regional atmospheric model and four patterns of large‐scale Southern Hemisphere climate variability: the southern baroclinic annular mode, the southern annular mode, and the two Pacific‐South American teleconnection patterns. Variations in all four patterns influence the spatial configuration of precipitation over Antarctica, consistent with their signatures in high‐latitude meridional moisture fluxes. They impact not only the mean but also the incidence of extreme precipitation events. Current coupled‐climate models are able to reproduce all four patterns of atmospheric variability but struggle to correctly replicate their regional impacts on Antarctic climate. Thus, linking these patterns directly to Antarctic precipitation variability may allow a better estimate of future changes in precipitation than using model output alone.

## Key Points

- The primary Southern Hemisphere extratropical circulation patterns all influence the spatial distribution of Antarctic precipitation
- They impact not only the mean but also the incidence of extreme precipitation events
- Locally, extreme precipitation may be associated especially with one polarity of a circulation pattern

## 1 Introduction

Model projections of future climate indicate that Antarctic precipitation is likely to increase over the remainder of the 21st century (e.g., Frieler et al., 2015; Ligtenberg et al., 2013; Palerme et al., 2016), as the air masses that deliver the majority of snow to the continent become warmer and thus able to increase their capacity to retain moisture. This will act to partially counteract the expected rise in global sea level due to thermal expansion and loss of terrestrial ice. However, despite its importance in the Earth system, a lack of direct observations have meant that Antarctic precipitation is relatively poorly constrained. Estimates have been derived from ice core data, atmospheric reanalyses, regional climate models, and satellite data (e.g., Behrangi et al., 2016; Lenaerts et al., 2012; Monaghan et al., 2006; Palerme et al., 2014). These suggest that there has been no clear increase in total Antarctic precipitation in recent decades (Monaghan et al., 2006), perhaps as expected given the lack of a consistent continent‐wide temperature trend (e.g., Turner et al., 2005) and high interannual snowfall variability (Lenaerts et al., 2012). Nonetheless, Frieler et al. (2015) used ice‐core data and model palaeo‐ and future‐climate simulations to derive a continent‐scale increase in accumulation of ~5 ± 1% K^−1^.

Monaghan and Bromwich (2008) showed that, while there may be no recent trend in total Antarctic precipitation, there have been opposing trends in different parts of the continent, some of which are statistically significant (cf., their Figure 6). The spatial distribution of precipitation across Antarctica results primarily from the interaction between the surface orography and patterns of atmospheric circulation variability. In coastal regions the majority of precipitation falls in association with the passage of synoptic‐scale weather systems. The steep coastal Antarctic topography means that precipitation falling below ~1,000 m is primarily orographic and is associated with vertical motion due to lifting over the coast itself and “blocked” near‐surface layers moving parallel to the coast (e.g., Orr et al., 2008). In contrast, over the interior of the high Antarctic Plateau the majority of the small amount of precipitation (in some areas <50 mm yr^−1^) comes from year‐round “clear‐sky” precipitation of ice crystals. However, occasionally, atmospheric blocking pushes weather systems inland onto the Plateau, where they cause rapid changes in weather and can contribute a significant proportion (~25%) of the annual accumulation over a few days (e.g., Massom et al., 2004). Hence, understanding the relationships between variations in the atmospheric circulation and Antarctic precipitation is critical for understanding the spatial distribution of precipitation across the continent.

The aim of this paper is to provide a first detailed analysis of how four principal patterns of large‐scale Southern Hemisphere (SH) circulation variability influence the spatial distribution of Antarctic precipitation. The atmospheric patterns comprise the southern baroclinic annular mode (BAM), the southern annular mode (SAM), and the two Pacific‐South American (PSA) teleconnection patterns. Previously, Marshall and Thompson (2016, hereafter MT16) analyzed the linkages between these circulation patterns and the spatial patterns of Antarctic near‐surface air temperatures (SATs) based on observations and reanalysis data. Here we provide analogous analyses for Antarctic precipitation.

## 2 Data and Methodology

### 2.1 Data

We use two primary sources: atmospheric data comes from (1) the European Centre for Medium Range Weather Forecasts (ECMWF) ERA‐Interim reanalysis data (Dee et al., 2011) for the 35 year period from 1979 to 2013, with the single exception of daily estimates of precipitation obtained by (2) the Regional Atmospheric Climate Model (RACMO2), forced by 6‐hourly ERA‐Interim data at its lateral boundaries: the sea surface temperature and sea ice fields used by the reanalysis are described by Dee et al. (2011, their Table 1). RACMO2 has been used to estimate recent and future Antarctic mass balance (e.g., Lenaerts et al., 2012; Ligtenberg et al., 2013; van de Berg et al., 2006; van Wessem et al., 2016). There are very few reliable precipitation measurements in Antarctica: thus, RACMO2 data have been validated against observations of surface mass balance (SMB), for which it has been found to outperform reanalyses in terms of representing the spatial patterns. Thus, given that precipitation is the dominant term in SMB across most of Antarctica, indirectly, this is a robust evaluation.

The model has a spatial resolution of 27 km and 40 vertical levels. Specifically, we use a version (2.3) in which additional upper air relaxation is employed (van de Berg & Medley, 2016). In previous versions of RACMO, the model was only adjusted at its lateral boundaries, while in version 2.3, the upper atmosphere is also weakly relaxed to ERA‐Interim, allowing the interannual variability in the reanalysis together with the superior surface climatology given by RACMO2. van de Berg and Medley (2016) concluded that interannual precipitation variability in the model is improved with the only downside being some smoothing of the precipitation field over the steep orography of the Antarctic Peninsula.

Daily‐mean values of the eddy meridional moisture flux at 850 hPa are found as *v* ^*^ *q* ^*^, where *v* is the meridional wind component, *q* is the specific humidity, and the asterisk denotes departures from the daily mean. We also use 10 m winds to demonstrate the impact that the patterns have on the near‐surface circulation.

The significance of differences between mean daily precipitation anomalies between positive and negative polarities of an index is calculated using the Kruskal‐Wallis test (Kruskal & Wallis, 1952). This is a nonparametric one way analysis of variance test on the ranked data and is employed because of the nonnormal distribution of the precipitation anomalies.

### 2.2 Patterns of Variability

The four patterns of atmospheric variability analyzed here are discussed in more detail in MT16. In brief, the BAM is derived as the time series of the leading empirical orthogonal function (EOF) of Southern Hemisphere (SH) daily mean zonal mean eddy kinetic energy while the SAM, PSA1, and PSA2 are defined as the first, second, and third EOFs of SH monthly mean 500 hPa geopotential height, respectively. The spatial domain used for the EOF analysis was 20–70°S. Daily mean time series of the SAM and PSA patterns are obtained by projecting daily mean 500 hPa geopotential height anomalies onto the respective EOF patterns.

The time series of all patterns are standardized so that their mean is zero and variance is one. We define days having a positive (negative) polarity of a particular index as those when the index is >1 (< −1): hereinafter, positive and negative polarities of a particular mode are indicated with a “+” or “−” subscript, respectively. By construction, the SAM and PSA patterns are not correlated with each other, and none are significantly correlated with the BAM (see MT16 for an explanation). Nevertheless, an individual day may be associated with the positive (and/or negative) polarity of more than one pattern at a time.

The BAM is characterized by hemispheric‐scale variations in the amplitudes of midlatitude storms, and its positive polarity is defined as days with larger than normal wave amplitudes (Thompson & Barnes, 2014; Thompson & Woodworth, 2014). MT16 found that the BAM has the smallest effect on Antarctic SATs of the four patterns considered here. The SAM is associated with meridional vacillations in the midlatitude jet, and its positive polarity is defined as a poleward displacement of the jet (e.g., Hartmann & Lo, 1998; Thompson & Wallace, 2000). There is a substantial literature of the impact of the SAM on Antarctic temperatures (e.g., Marshall, 2007; MT16) but relatively little previous analysis of its effect on precipitation (e.g., Genthon et al., 2003). A modeling study by van den Broeke and van Lipzig (2004) showed that the positive polarity of the SAM is linked to significant increases (decreases) in precipitation over the western Antarctic Peninsula (western Marie Byrd Land and the Ross Ice Shelf and parts of East Antarctica).

**Figure 1:**
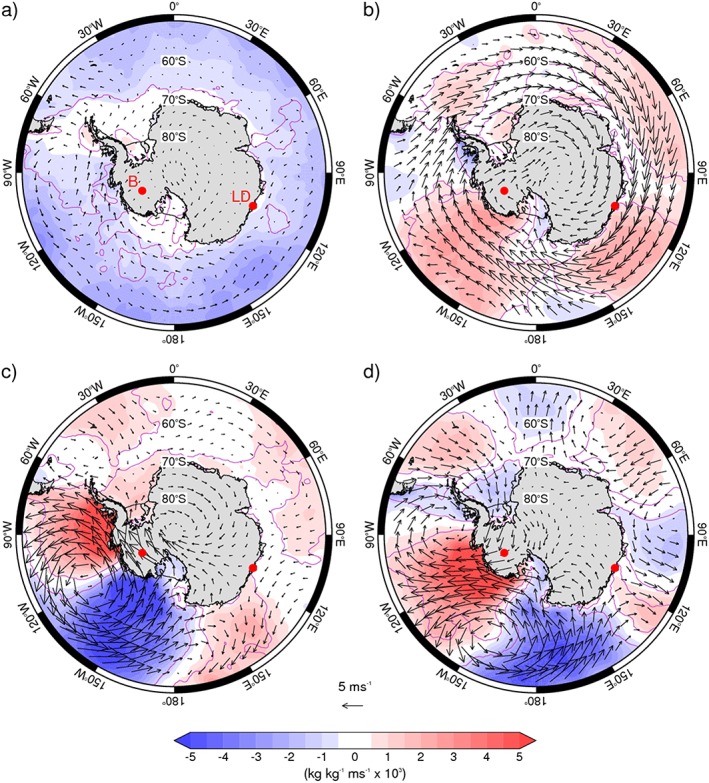
Differences in mean daily meridional moisture flux anomalies (*v* * *q* *) at 850 hPa (shading) and near‐surface wind anomalies (vectors) between the positive and negative polarities of (a) the BAM, (b) the SAM, (c) the PSA1 pattern, and (d) the PSA2 pattern. Negative (blue shading) denotes anomalously poleward moisture fluxes. In Figure 1a “B” and “LD” indicate the Byrd and Law Dome ice core sites, respectively, which are indicated as red circles in other figures.

The two PSA patterns (termed PSA1 and PSA2) are characterized by zonally asymmetric pressure anomalies that extend southeastward across the extratropical Pacific Ocean to the Southern Ocean off West Antarctica, and their positive polarities are indicated in Figure 1 (Mo & Higgins, 1998; Mo & Paegle, 2001). The PSA1 and PSA2 patterns are coupled with variations in tropical convection in the western and central Pacific, respectively: PSA1 is thus associated with broadscale El Niño–Southern Oscillation climate variability. Unsurprisingly, MT16 demonstrated that the two PSA modes had their largest impact on Antarctic SATs in West Antarctica, associated with strong anomalies in meridional heat fluxes in and out of the continent, but are also allied to SAT changes across parts of East Antarctica.

## 3 Results

To provide physical context for the linkages between large‐scale climate variability and Antarctic precipitation, we first explore the differences in the 850 hPa meridional moisture flux and near‐surface winds between the positive and negative polarities of the four patterns of atmospheric variability. Note that negative values (blue shading) denote poleward fluxes, and vice versa (Figure 1).

The BAM is linked to variations in eddy amplitudes in the SH atmospheric circulation but not the zonal mean flow. As such, it has a relatively weak signal in the near‐surface circulation but a notable signature in the poleward moisture flux across most of the Southern Ocean (the total moisture flux is dominated by its eddy component). The moisture fluxes associated with the positive polarity of the BAM (i.e., BAM_+_) are uniformly poleward and peak at midlatitudes (Figure 1a). SAM_+_ is linked to stronger circumpolar westerlies along ~60°S and, in general, equatorward moisture fluxes: exceptions include the region west of the Antarctic Peninsula, where there are strong onshore winds, and the western part of the Ross Sea (Figure 1b).

**Figure 2:**
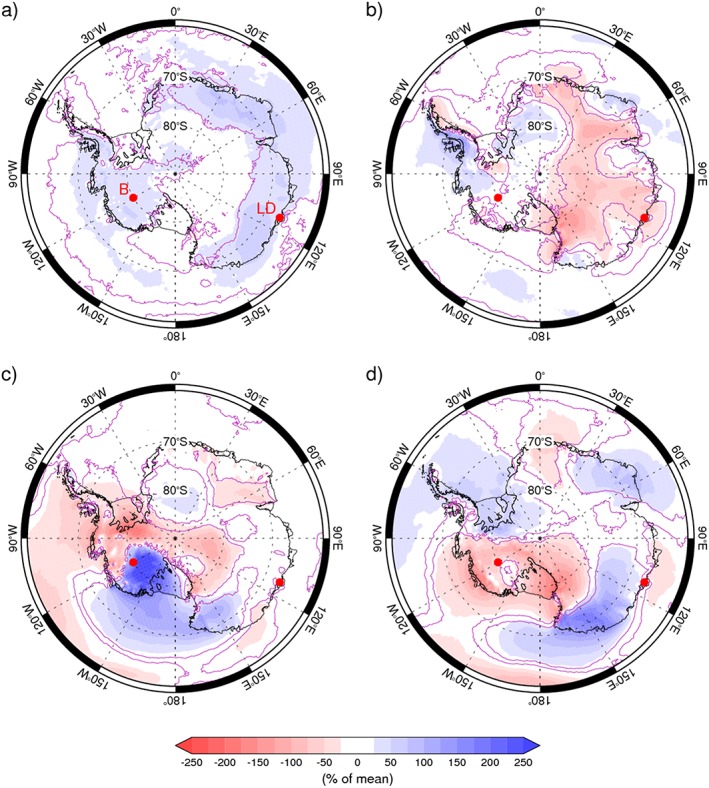
As Figure 1 but for the differences in mean daily precipitation anomalies. The purple contour represents regions where the difference is significant at the *p* < 0.01 level based on the Kruskal‐Wallis test.

The PSA patterns are dominated by circulation anomalies associated with an anticyclonic pressure anomaly off West Antarctica, which for PSA1 (PSA2) is centered at 120°W (150°W). Strong meridional wind anomalies bring especially large moisture fluxes into (out of) the continent to the west (east) of the high‐pressure anomaly. We note that in some areas of West Antarctica, PSA1_+_ and PSA2_+_ are associated with moisture flux anomalies of opposite sign, including Marie Byrd Land and the western Antarctic Peninsula. Elsewhere over the Southern Ocean, PSA1 has relatively little impact, with some regions having weak equatorward moisture flux anomalies, while PSA2 has a greater impact in the Eastern Hemisphere, with evidence of alternate bands of poleward and equatorward moisture flux anomalies. In general, the spatial pattern of the moisture flux anomalies in Figure 1 closely matches the eddy heat flux anomalies shown in Figure 2 of MT16, although there are local differences, particularly in coastal regions.

Difference plots of daily precipitation anomalies between positive and negative polarities of the four patterns of atmospheric circulation variability are shown in Figure 2. Individual seasons are shown in the supporting information (Figures S1–S4). For the most part, the seasonally varying results are very similar to those shown in Figure 2, with the most pronounced differences limited to the Plateau, where total precipitation amounts are small.

As indicated in Figure 2a, BAM_+_ is associated with significant increases in precipitation across much of coastal East Antarctica, West Antarctica, and the Antarctic Peninsula (blue shading), coincident with the anomalously poleward moisture fluxes across most of the Southern Ocean (Figures 1a). The seasonally stratified results indicate that the areas of increased precipitation are reduced somewhat in spring (Figure S1).

Figure 2b reveals that SAM_+_ is linked to decreased precipitation over much of the Antarctic Plateau, especially during fall (Figure S2a). There are areas of increased precipitation around parts of coastal East Antarctica, primarily in spring (Figure S2c), and, in particular, the western Antarctic Peninsula. In these locations the increase in the (north) westerly wind component enhances the advection of moisture toward the coast (Figure 1b) and the subsequent formation of orographic precipitation as the cooling air reaches saturation. The significant orographic barrier of the Antarctic Peninsula acts to form a precipitation shadow on the eastern side such that SAM_+_ leads to reduced precipitation here. Such detail has previously been observed in an older version of RACMO with a 14 km resolution (van Lipzig et al., 2008) but not at 55 km (van den Broeke & van Lipzig, 2004). Uotila et al., (2013) noted that SAM_+_ is associated with southward cyclone motion and increased cyclogenesis, especially between 110 and 170°E. However, Figure 2b indicates that this does not lead to greater precipitation in this region.

The composite difference for Antarctic daily precipitation between PSA1_+_ and PSA1_−_ is shown in Figure 2c and demonstrates an area of significant increase in precipitation associated with the former: the difference between the two polarities exceeds 2.5 times the mean value of precipitation over the Marie Byrd Land region of West Antarctica and the eastern part of the Ross Ice Shelf, reflecting the strong onshore moisture flux into this area during PSA1_+_ (Figure 1c). PSA1_+_ is also linked to reduced precipitation across the remainder of West Antarctica and most of the Antarctic Peninsula, consistent with Irving and Simmonds (2016) and Guo et al., (2004), who explored the correlations between El Niño and precipitation in this area. There are small regions of both significant increases and decreases in precipitation across East Antarctica associated with PSA1_+_, the latter predominantly in spring and summer (Figures S3c/S3d), but overall a change in the polarity of the PSA1 pattern has less impact on precipitation in this region of the continent than for the other circulation patterns studied here.

In contrast to PSA1_+_, precipitation during PSA2_+_ tends to be reduced over most of West Antarctica and increased over the Antarctic Peninsula: this results from the westward shift in the position of the anticyclonic center that dominates the circulation anomalies linked to PSA2_+_ as compared to those associated with PSA1_+_ (cf. Figures 1c and 1d). The circulation anomalies connected to PSA2 around coastal East Antarctica lead to significant precipitation anomalies of both signs across most of this region (Figure 2d), which are broadly consistent across all seasons (Figure S4).

Many of the key features identified in Figure 2 are statistically significant. However, it is important to note that—at least on daily time scales—variations in the large‐scale atmospheric circulation account for relatively modest fractions of the total day‐to‐day variability in precipitation. For example, Figure S5 summarizes the components of the total daily precipitation variability accounted for by each of the four patterns across Antarctica. Unsurprisingly, the areas where the differences between the two polarities of a mode are greatest tend to be where it contributes most to the total precipitation variability (cf. Figures 2 and S5). However, comparing the proportion of the total daily standard deviation of precipitation (Figure S5a) associated with the various patterns (Figures S5b–S5e), it is clear that much of the daily precipitation variability is connected to either other forms of variability or local weather “noise”, with orography clearly playing a major role in the Antarctic coastal regions. The greatest proportion of daily precipitation variability linked to one of the patterns is for PSA1 in West Antarctica, where it contributes ~40% of the total.

**Figure 3:**
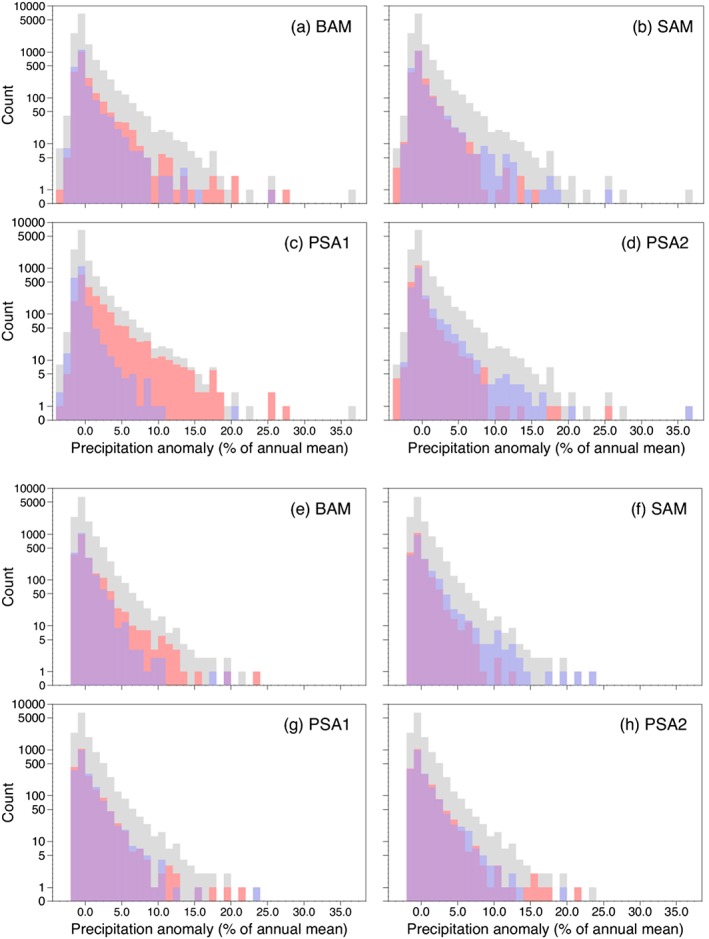
Probability distribution functions (pdfs) of daily precipitation anomalies in percentage bins of the annual mean at Byrd (grey) subdivided between days having positive (red) and negative (blue) polarities for (a) the BAM, (b) the SAM, (c) the PSA1 pattern, and (d) the PSA2 pattern. Similarly, for Law Dome for (e) the BAM, (f) the SAM, (g) the PSA1 pattern, and (h) the PSA2 pattern. The purple area is where the two pdfs overlap.

Figure 2 indicates the differences in daily‐mean precipitation between the two polarities of the circulation indices, but it does necessarily reveal the extent to which the patterns are associated with changes in extreme precipitation, since they do not account for the skewed distribution of daily precipitation. Therefore, we produced separate probability distribution functions for the positive and negative polarities of the four circulation indices analyzed at two sites where ice cores have been drilled. There have been a number of studies using Antarctic ice core accumulation as a proxy for past variability in atmospheric circulation indices (e.g., Thomas et al., 2008; van Ommen & Morgan, 2010), so it is especially important to understand the extent to which extreme precipitation events may influence the signal at these sites. Here we show results for Byrd in West Antarctica (Figures 3a–3d), which until recently was one of the fastest warming regions on Earth (Bromwich et al., 2013), and Law Dome in East Antarctica (Figures 3e–3h), which has a strong maritime influence (van Ommen & Morgan, 2010).

At Byrd, days with greater than average precipitation are more likely to be associated with BAM_+_, SAM_−_, and especially PSA1_+_ and PSA2_−_. The incidence of higher than normal precipitation during PSA1_+_ is particularly striking. However, we note that the highest daily RACMO precipitation, which took place on 29 May 1998 and was ~0.11 m water equivalent (+36.6% anomaly in terms of the annual mean; cf. Figures 3a–3d), occurred during PSA2_−_ conditions (Figure 3d). The third highest daily precipitation actually occurred during PSA2_+_, in addition to PSA1_+_ and BAM_+_. In general, the days with the most negative precipitation anomalies (i.e., those without precipitation) tend to be associated with the opposite polarity to those days with the highest precipitation.

For Law Dome, higher precipitation days are also more likely to be linked to BAM_+_ and SAM_−_ while there is relatively little difference in precipitation between the two polarities of both PSA patterns, as also indicated by Figures 2c and 2d. van Ommen and Morgan (2010) previously showed that reduced precipitation at Law Dome was associated with a negative SAM index based on annual accumulation at the site. In terms of extremes in daily precipitation, Figure 3f demonstrates that four of the six highest values occurred during SAM_−_ conditions while none of the top 20 highest occurred during SAM_+_. In contrast, days of both polarities of the other three circulation indices exist in the 5 days of greatest precipitation. During 12 July 2009, when the maximum daily RACMO precipitation of ~0.27 m water equivalent fell (+23.1% anomaly in terms of the annual mean; cf. Figures 3e–3h), there were BAM_+_, SAM_−_, and PSA1_−_ conditions (Figures 3e–3g). There is relatively little difference in the likelihood of days with zero precipitation at Law Dome being associated with a particular polarity of one of the circulation patterns.

## 4 Discussion and Conclusions

We provide the first comprehensive analysis of the role that the four principal patterns of Southern Hemisphere high‐latitude circulation variability—the BAM, SAM, and two PSA patterns—have on the spatial distribution of precipitation across Antarctica. Using composite difference plots (Figure 2), we demonstrate the following

1. All four patterns influence the spatial patterns of Antarctic precipitation.
2. The positive polarity of the BAM (anomalously high storm amplitudes over the Southern Ocean) leads to greater precipitation over coastal East Antarctica.
3. The positive polarity of the SAM results in enhanced precipitation across much of West Antarctica and the western Antarctic Peninsula. However, the enhanced westerly winds impinging on the steep orography cause a “precipitation shadow” on the lee side of the Peninsula. SAM_+_ also acts to reduce the relatively small amounts of precipitation over the Plateau.
4. The principal impact of the PSA patterns on precipitation anomalies is observed in West Antarctica, where significant increases (decreases) are linked with strong meridional moisture fluxes into (out of) the continent. However, both PSA patterns, especially PSA2, are also linked with precipitation anomalies across other parts of Antarctica.

We examined pdfs of the daily time series of precipitation at Byrd and Law Dome, two sites where well‐studied ice cores have been drilled, to understand whether the circulation patterns are responsible for the most significant precipitation anomalies (Figure 3). At the former site, days with the most precipitation were strongly associated with PSA_+_ and, to a lesser extent, SAM_−_ and PSA2_−_ conditions. In contrast, at Law Dome, days with higher precipitation are typically allied to BAM_−_ and SAM_+_.

By comparing our results (Figure 2) with those of MT16 (their Figure 3) we can ascertain whether a precipitation anomaly linked to a particular circulation mode is associated with warmer or colder than average SAT across Antarctica. For the two PSA modes and the majority of the anomaly patterns linked to the SAM, greater precipitation is coupled with warmer SATs (and vice versa). This is unsurprising given that the principal circulation anomalies connected with the PSA patterns are related to strong meridional flow into or out of the continent, which, if coming from the north, will bring warm moist oceanic air into the continent. A clear positive relationship between precipitation and SAT is also observed at longer time scales in relation to the longitude of the climatological Amundsen Sea Low off West Antarctica (Hosking et al., 2013). The regions of Antarctica where precipitation and SAT are not both positively correlated together in relation to SAM variability include small regions of both West and East Antarctica, also noted by van den Broeke and van Lipzig (2004), and the eastern side of the Antarctic Peninsula: here the steep orography means that the warm westerly winds associated with SAM_+_ conditions lead to a reduction in precipitation on the lee side (e.g., van Lipzig et al., 2008). In contrast, BAM_+_ is linked to colder SATs in combination with greater precipitation across much of coastal Antarctica, although anomalies in both parameters are relatively small and not especially robust (MW16).

The long‐term trends in the circulation over Antarctica have been toward a greater frequency of both the positive polarity of the SAM during austral summer (e.g., Marshall, 2007; Thompson & Solomon, 2002) and conditions reminiscent of the negative polarity of PSA1 that are tied to sea surface temperature anomalies in the tropical Pacific (e.g., Ding et al., 2011; Feng et al., 2013; L'Heureux et al., 2013). Looking at the trends in Antarctic snowfall given by Monaghan and Bromwich (2008, Figure 6), one can argue that the largest and/or most significant trends in snowfall can be related to these trends in atmospheric circulation. For example, the dipole of marked increase (decrease) in eastern (western) West Antarctica is the opposite of Figure 2c, suggesting a role for PSA1_−_ conditions.

However, during the 35 year period studied here, there are no statistically significant trends in the four circulation patterns in the *annual mean*. Seasonally, there is a positive trend in the BAM in fall (March‐April‐May) at *p* < 0.10 and in the SAM in summer (December‐January‐February) at *p* < 0.05. Plots of trends in daily precipitation in these two seasons and the fraction of the trends that are congruent with the circulation patterns are provided in the supporting information. Figure S6 indicates that the weak trend in the BAM has had little impact on the overall precipitation trend during the fall. In contrast, Figure S7 reveals that the greater frequency of SAM_+_ days during summer is predominantly responsible for the opposing trends in precipitation on the two sides of the Antarctic Peninsula, a region where the trends in the SAM are also known to have had a significant impact on SAT in this season (Marshall et al., 2006).

Climate model projections forecast an increase in Antarctic precipitation as global temperatures rise. Indeed, modeling studies suggest that a distinct pattern of austral summer drying and moistening observed in Southern Hemisphere midlatitudes and high latitudes, respectively, over the last few decades is likely to have a strong anthropogenic component (Fyfe et al., 2012). If the patterns of circulation variability examined here change on low‐frequency time scales—as is the case for the SAM and to a lesser extent the PSA patterns—there will be a response in the associated precipitation pattern, which in turn will have an impact on the regional surface mass balance. Current global coupled‐climate models, which are employed to make projections of future climate change, are generally able to reproduce these atmospheric patterns but struggle with correctly replicating their regional impacts on Antarctic climate (e.g., Marshall & Bracegirdle, 2015). Therefore, an examination of how the main patterns of atmospheric circulation are likely to change in the future may prove more useful in predicting how regional‐scale Antarctic precipitation will evolve than a direct analysis of changes in modeled precipitation itself.

## Supporting information



Supporting Information S1
