# Supplementary material for: The Signature of Southern Hemisphere Atmospheric Circulation Patterns in Antarctic Precipitation
Source: Geophys Res Lett. 2017 Nov 18;44(22):11580–9. doi: 10.1002/2017GL075998 (PMC5784396; doi:10.1002/2017GL075998)
Supplement: Supplementary file 1 — Supporting Information S1 [file GRL-44-11580-s001.pdf]

Supporting Information for

The signature of Southern Hemisphere atmospheric circulation patterns in Antarctic precipitation

Gareth J. Marshall[1]

David W. J. Thompson[2]

Michiel R. van den Broeke[3]

[1]British Antarctic Survey

[2]Department of Atmospheric Science, CSU

[3]Institute for Marine and Atmospheric Research, Utrecht University

Contents of this file

Figures S1 to S7

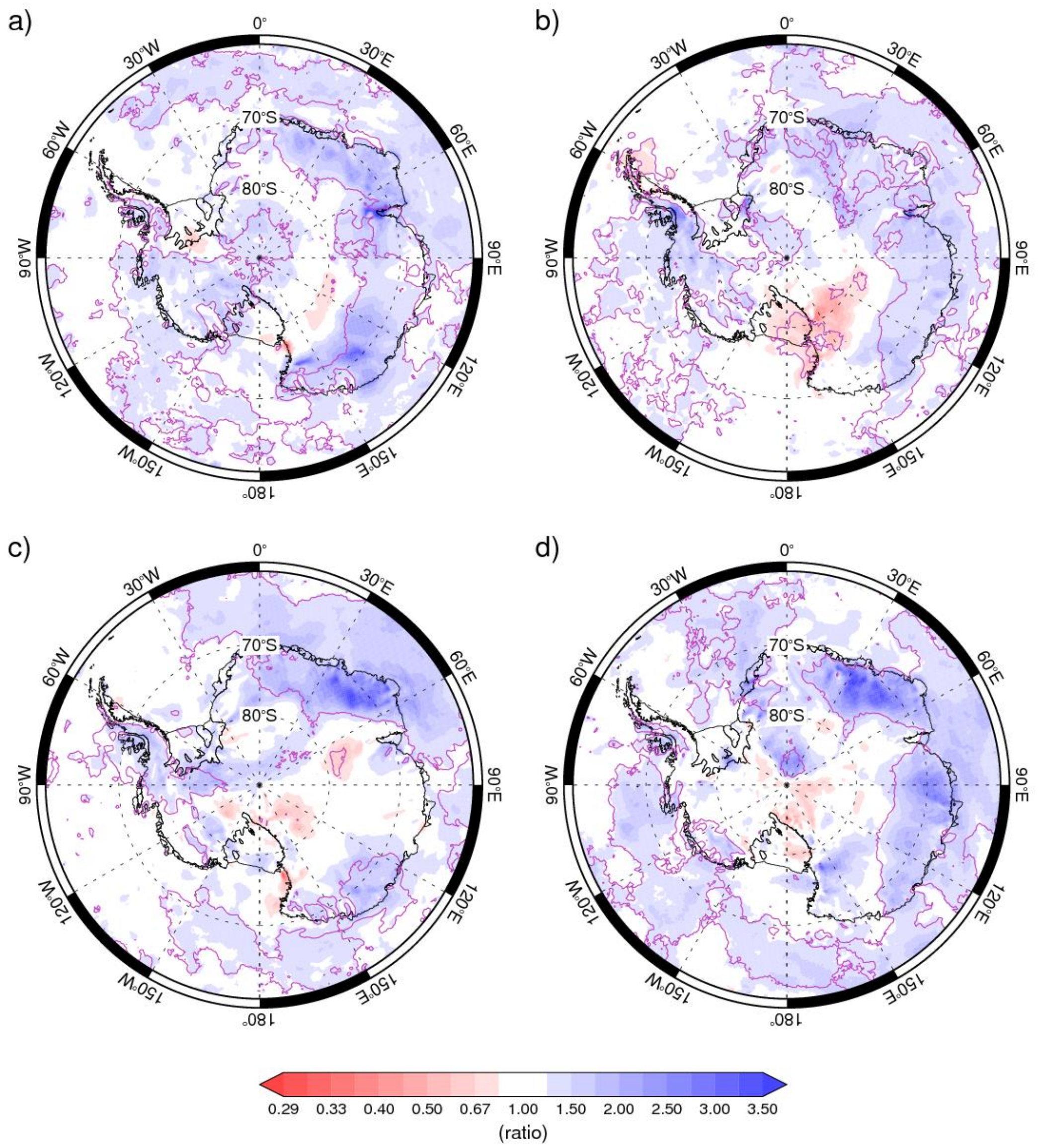

Figure S1. Differences in mean daily precipitation anomalies between the positive and negative polarities of the BAM for (a) fall (March-April-May), (b) winter (June-July-August), (c) spring (September-October-November), and (d) summer (December-January-February). The purple contour represents regions where the difference is significant at the $p < 0.01$ level based on the Kruskal-Wallis test.

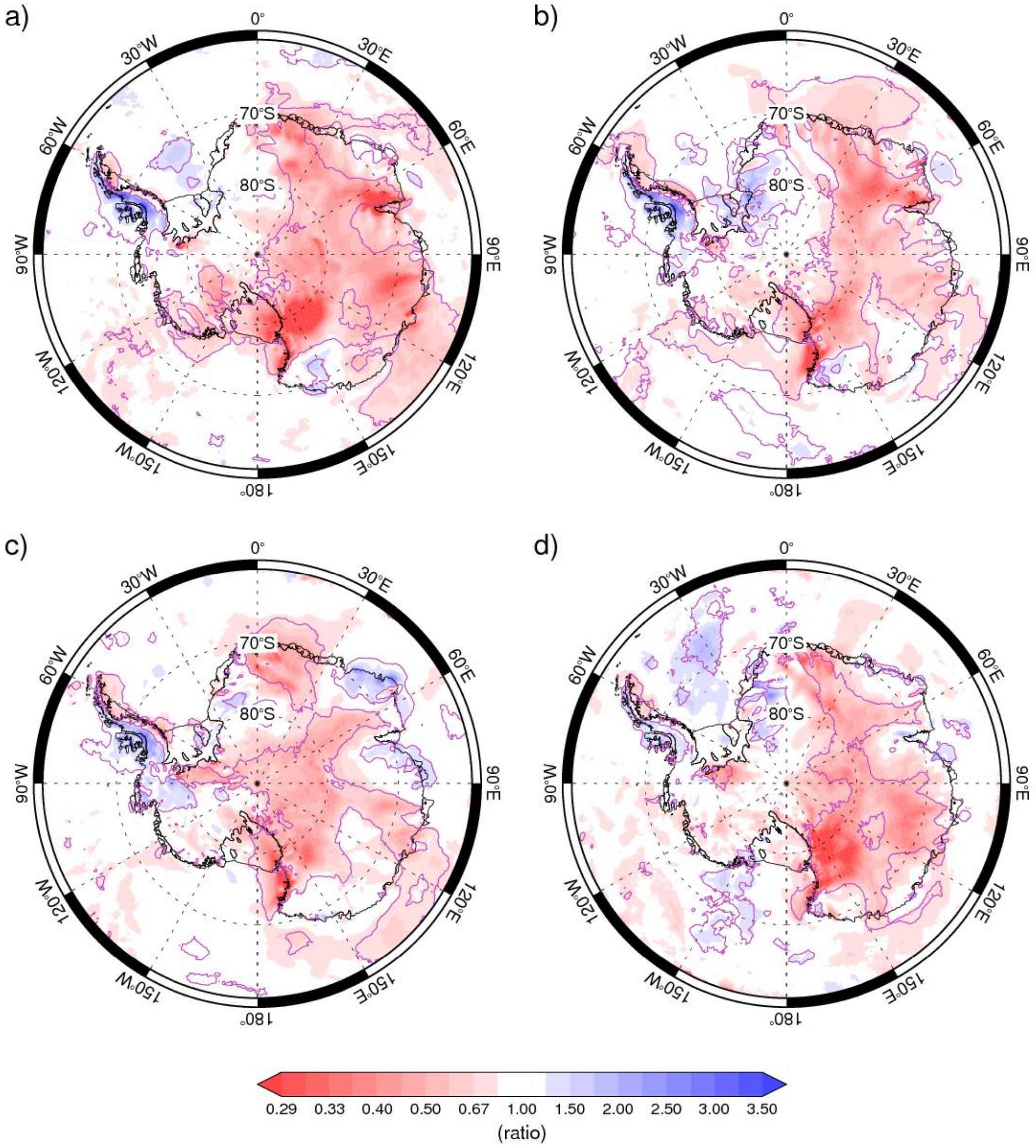

Figure S2. As Figure S1 but for the SAM.

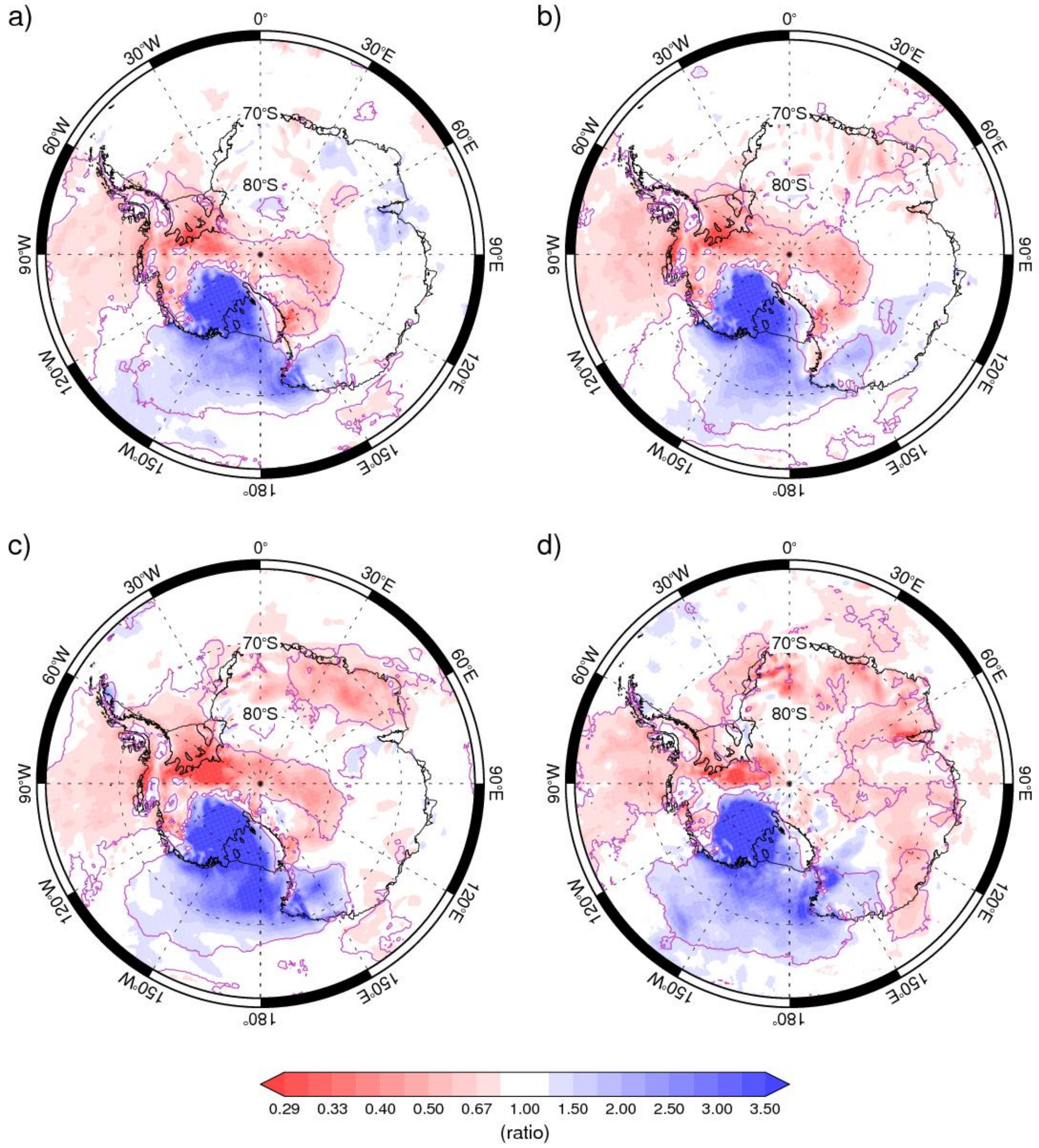

Figure S3. As Figure S1 but for the PSA1 pattern.

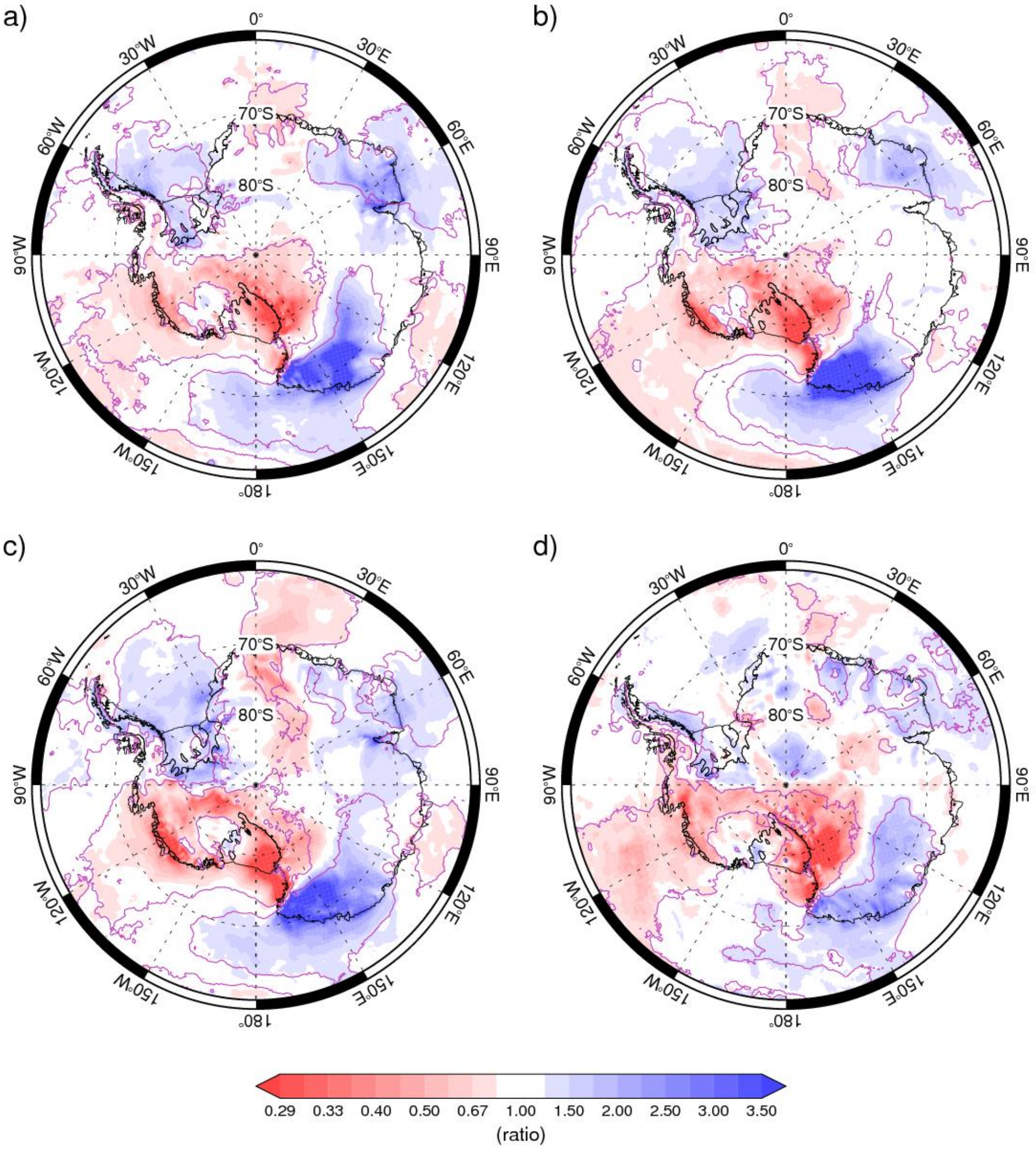

Figure S4. As Figure S1 but for the PSA2 pattern.

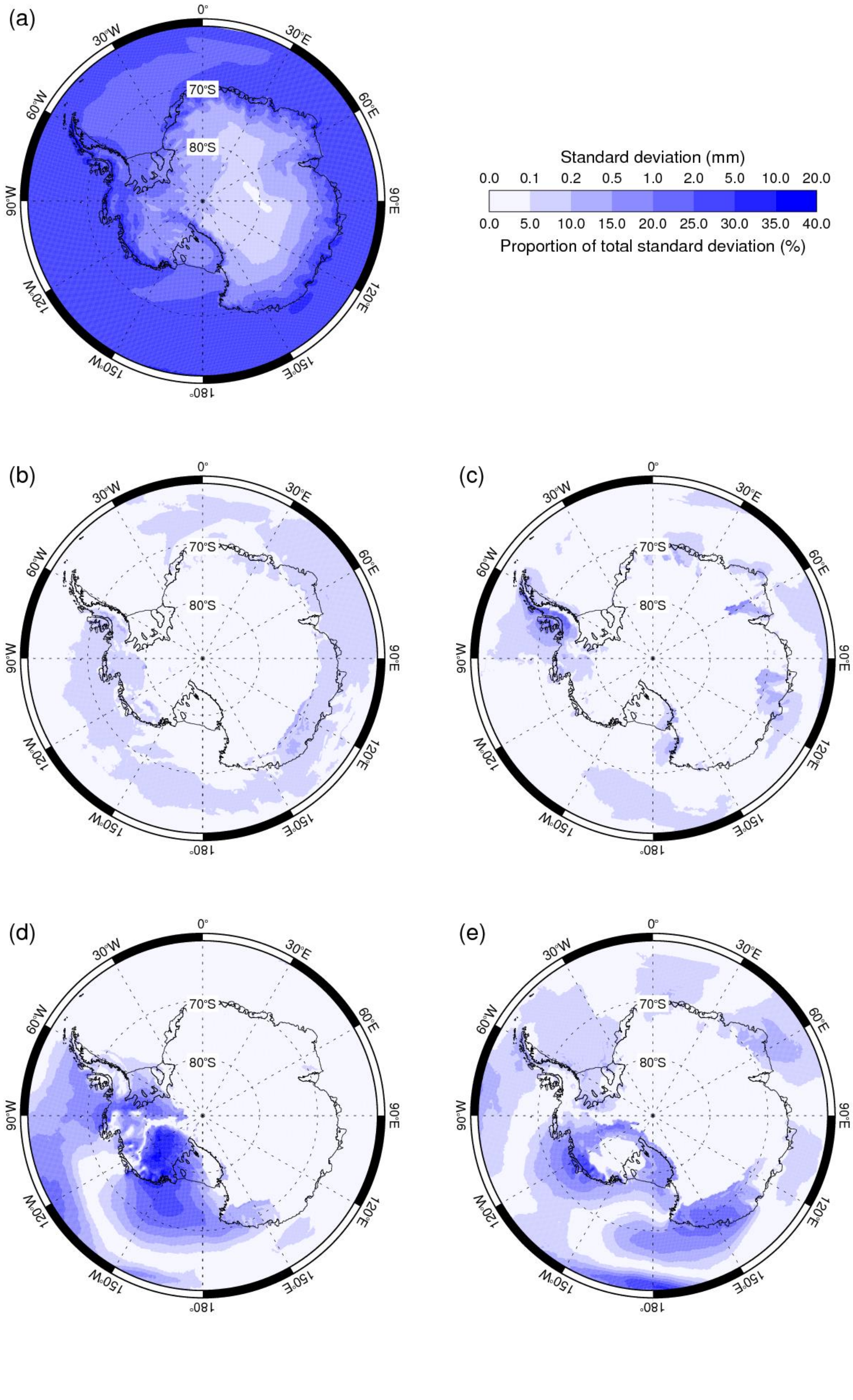

Figure S5. Standard deviation of daily Antarctic precipitation: (a) all days, the proportion of (a) associated with (b) the BAM, (c) the SAM, (d) the PSA1 pattern, and (e) the PSA2 pattern.

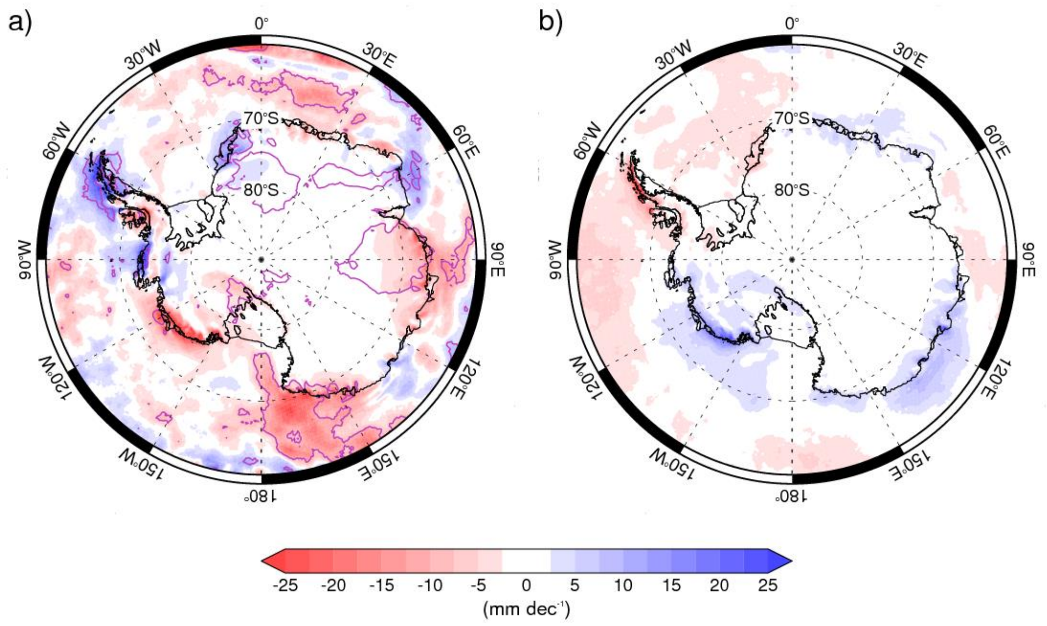

Figure S6. Fall precipitation trend, 1979-2013 (a) total, and (b) congruent with the BAM. The trend congruent with the BAM was calculated as the fall precipitation trend (a) multiplied by the regression coefficient between the detrended BAM and the detrended seasonal precipitation data. The purple contour represents regions where the trend is significant at the $p < 0.10$ level.

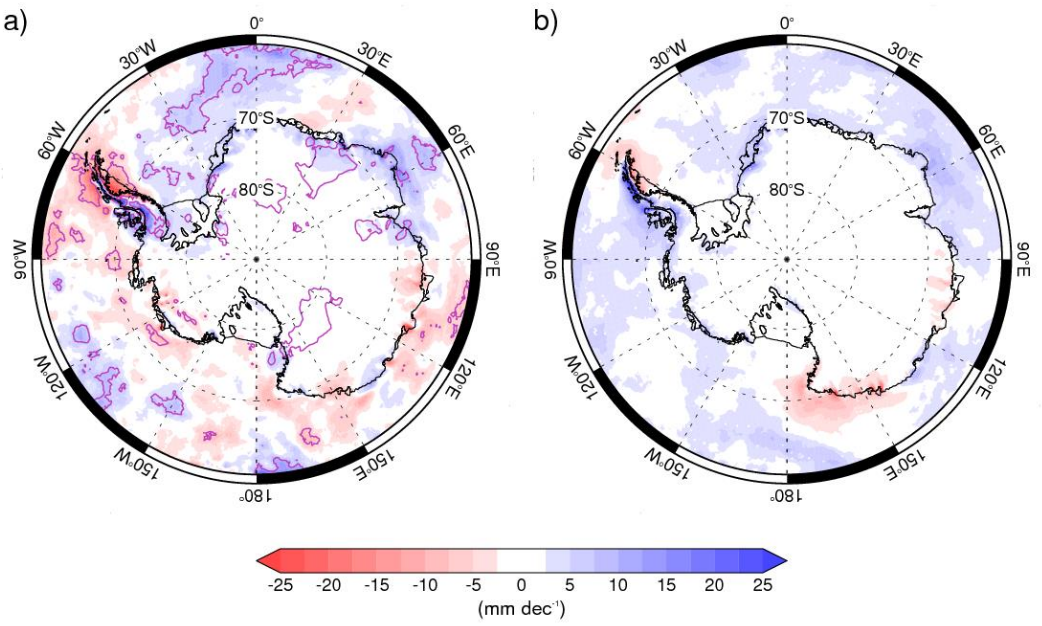

Figure S7. Summer precipitation trend, 1979-2013 (a) total, and (b) congruent with the SAM. The trend congruent with the SAM was calculated as the summer precipitation trend (a) multiplied by the regression coefficient between the detrended SAM and the detrended seasonal precipitation data. The purple contour represents regions where the trend is significant at the $p < 0.10$ level.
